# Synthesis of a [2.2.2] Cryptand Containing Reactive Functional Groups

**DOI:** 10.3390/molecules14093600

**Published:** 2009-09-15

**Authors:** Yousry M.A. Naguib

**Affiliations:** Faculty of Education, Suez Canal University, El-Arish, Egypt; Email: yousryn@gmail.com

**Keywords:** dibenzocryptand [2.2.2], ionophore, potassium

## Abstract

The functional group-containing potassium ionophore 19^5^,24^5^-dibromo-4,7,13,16,20,23-hexaoxa-1,10-diaza-19(1,2),24(1,2)-dibenzabicyclo[8.8.6]tetracosaphane has been synthesized.

## Introduction

Potassium (K^+^) is a major cationic constituent of living cells; its concentration in intercellular living cells is around 150 mmol·dm^-3^, whilst its extracellular levels are typically around 4 mmol·dm^-3^. Potassium level measurements of are importance in the fields of cell physiology and clinical medicine [1,2]. Fluoroionophores based on a fluorescent coumarin group probe and the ionophore 1,10-diaza-18-crown-6 chelating group (abbreviated by the acronym CD18), which is selective for K^+^ ions, and capable of measuring K^+^ in an aqueous physiological environment have been reported [3].

Fluoroionophore sensors possess a guest binding site (ionophore) having a heteroatom with nonbonding electron pairs such as nitrogen, and a photon-interaction site (fluorophore) capable of accepting electrons. In the absence of a cation that binds selectively in the cavity of ionophore, when the fluorophore is excited by a photon, electron transfer from the ionophore to the fluorophore results in fluorescence quenching. In the presence of a cation which selectively binds to the ionophore and hence inhibits the electron transfer process, the fluorescence of fluorophore is switched on.

In order to attain high, yet selective binding of a potassium ion chelator some rigidity in the system was considered necessary compared to the selective binder CD18. Hence, the 1,10-diaza-18-crown-6 chelating group in CD18 was replaced by the bicyclic ionophore “cryptand [2.2.2]” which resulted in a selective, high affinity fluoresecent probe for K^+^ abbreviated CD222 [4]. 

Cryptands are cavities containing macromolecules which form stable complexes with alkali metal ions. For a given cation, the stability constant is largest for the cation which fits best into the cavity of the ligand. Thus stability maxima are found for Li[2.1.1]^+^, Na[2.2.1]^+^, and K[2.2.2]^+^, respectively [5]. A similar fluoroionophore based on the ionophore cryptand [2.2.2] (abbreviated MCC) for measuring potassium, namely, 6,7-(4-methyl)coumaro-[2.2.2] cryptand, was also reported [6]. Other potassium fluoroionophores based on triazocryptands have been reported [7, 8]. The cavity size of the cryptand [2.2.2] (2.8 Å in diameter) closely matches the size of potassium cation (2.66 Å). This paper describes the synthesis of a bromo-derivative of the ionophore dibenzo-cryptand [2.2.2] which serves as a precursor of potential fluoroionophores for potassium ions [9]. The fluorophore can be introduced by, for example, reacting the bromodibenzocryptand [2.2.2] with *t*-butyl lithium, followed by reaction with a 2,7-dichloroxanth-9-one derivative to give a fluoroscein type fluoroionophore.

## Results and Discussion

**Scheme 1:**
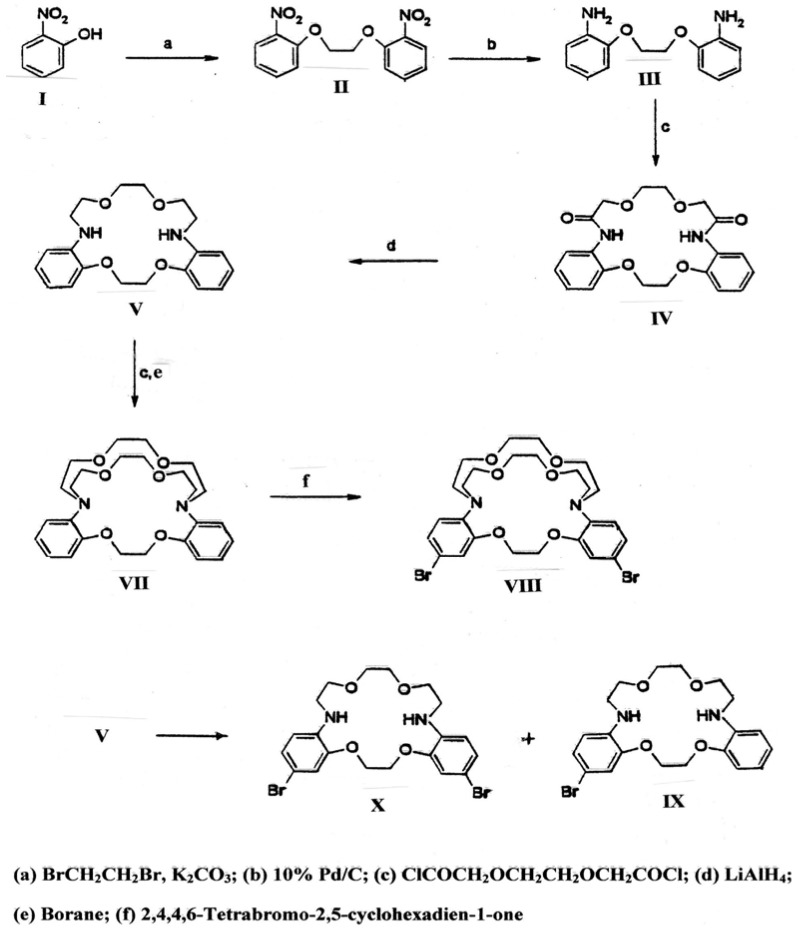
Synthesis of bromodibenzocryptand [2.2.2].

The synthesis of the dibenzocryptand [2.2.2] derivative; namely 19^5^,24^5^-dibromo-4,7,13,16,20,23-hexaoxa-1,10-diaza-19(1,2),24(1,2)-dibenzabicyclo[8.8.6]tetracosaphane (**VIII**) is outlined in Scheme 1. The commercially available compound 2-nitrophenol (**I**) was chosen as a starting material.

**Figure 1:**
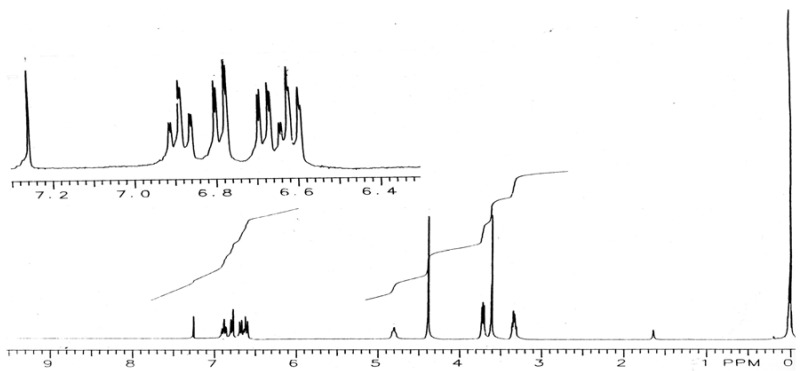
^1^H-NMR (300 MHz, CDCl_3_) of **V**.

Treatment of two equivalents of (**I**) with 1,2-dibromoethane and potassium carbonate in dimethyl formamide (DMF) afforded 1,2-bis(2-nitrophenoxy)ethane (**II**). Reduction of **II** with 10% palladium-on-charcoal as the catalyst produced the diamino derivative **III**. The diamine **III** was next reacted in tetrahydrofuran under high dilution conditions [10] with 3,6-dioxaoctanedioyl dichloride (1,2-ethylene-*O,O*-diglycolic acid chloride) to gave the lactam **IV**. The lactam **IV** was reduced with lithium aluminum hydride (LiAlH_4_) in THF to give the azacrown **V** [11]. Figure 1 shows the complete ^1^H-NMR spectrum of **V** and the insert shows the 6.4–7.2 ppm region, and the assignment of spectral data are given in the Experimental section.

Subsequent treatment of **V** with 1,2-ethylene-*O,O*-diglycolic acid chloride gave **VI** which upon reduction with diborane in tetrahydrofuran [12] furnished the cryptand **VII**. Calo *et al*. have reported a simple method for monobromination of aromatic amines predominantly or exclusively in the *para*-position utilizing 2,4,4,6-tetrabromo-2,5-cyclohexadien-1-one [13]. A similar procedure is also described in Organic Syntheses [14] for the preparation of *para*-brominated aromatic amines in high yield, for example bromination of *ortho*-anisidine (closely related to compounds **V** and **VII**) with 2,4,4,6-tetrabromo-2,5-cyclohexadien-1-one yielded 85% of the corresponding *para*-bromo-derivative [14]. These data support the expected *para*-bromination with 2,4,4,6-tetrabromo-2,5-cyclohexadien-1-one of compound **VII** to the dibromocryptand **VIII**, and of **V** to **X**. 

**Figure 2:**
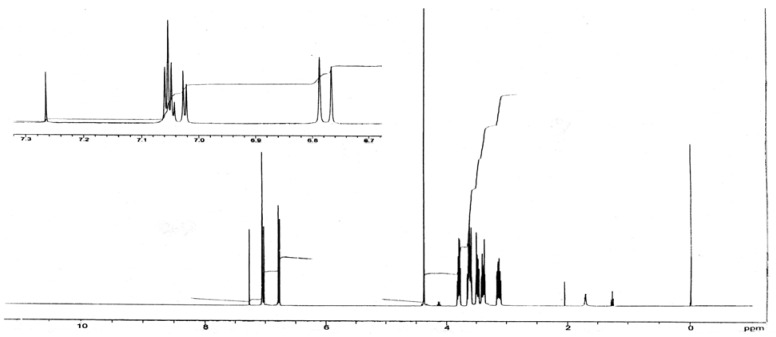
^1^H-NMR (400 MHz, CDCl_3_) of **VIII**.

**Figure 3:**
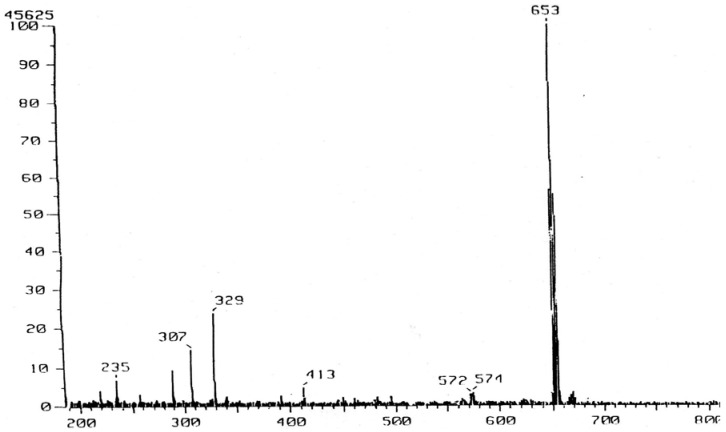
Mass spectrum (FAB) of **VIII**.

Figure 2 shows the complete ^1^H-NMR spectrum of **VIII** and the insert shows the 6.7–7.3 ppm region. Figure 3 shows the FAB (fast atom bombardment) mass spectrum of **VIII**, which displays a [M+Na]^+^ ion at *m/z* 651 (based on ^79^Br) and this gives M = 628, consistent with molecular formula of **VIII**. The isotope pattern reflects a dibromo composition. The ions at *m/z* 307 and 329 are [2M+H]^+^ and [2M+Na]^+^ respectively for the nitrobenzyl alcohol used as the FAB matrix.

**Figure 4:**
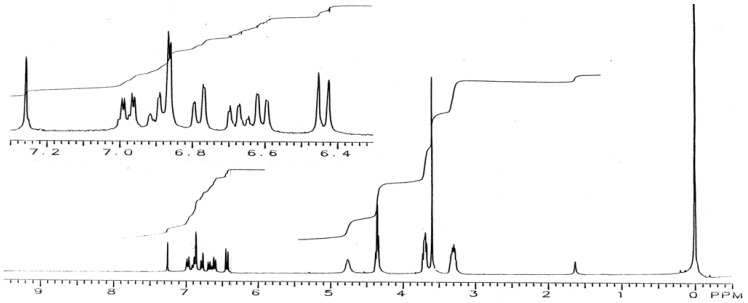
^1^H-NMR (300 MHz, CDCl_3_) of **IX**.

**Figure 5:**
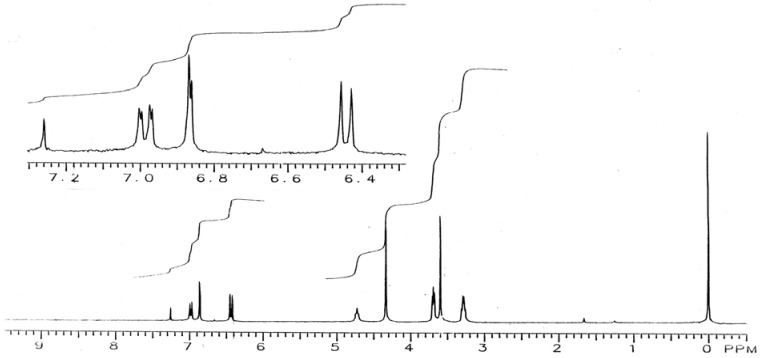
^1^H-NMR (300 MHz, CDCl_3_) of **X**.

The dibromocryptand **VIII** was also prepared by an alternative reaction sequence starting with azacrown **V**. Bromination of **V** with bromine afforded both monobromo azacrown **IX** and dibromo azacrown **X**. Compound **X** was also prepared by the treatment of diamine **V** with 2,4,4,6-tetrabromo-2,5-cyclohexadien-1-one. Figure 4 and Figure 5 show the ^1^H-NMR spectra of IX and X, respectively. Treatment of **X** with 3,6-dioxaoctanedioyl dichloride afforded **XI** (19^5^,24^5^-Dibromo-4,7,13,16,20,23-hexaoxa-1,10-diaza-19(1,2)24(1,2)-dibenzabicyclo[8.8.6]tetracosaphane-2,9-dione) which upon reduc-tion with borane in THF gave the dibromocryptand **VIII**.

The ^1^H-NMR compound **X** (Figure 5) displays signals at δ 6.44 (d, *J* = 8.7 Hz, 2H, 1^3^-H, 6^3^-H), 6.86 (d, *J* = 2 Hz, 2H, 1^6^-H, 6^6^-H), 6.99 (dd, *J* = 2 and 8.7 Hz, 2H, 1^4^-H, 6^4^-H) assigned to 1^5^,6^5^-dibromo-2,5,10,13-tetraoxa-7,16-diaza-1(1,2),6(1,2)-benzenecyclohexadecaphane. Comparison of the spectral data of **X** with those of the diamine **V** reveals that bromination *para*- to the amino group results in an upfield shift (0.18 ppm) of 1^3^-H (from 6.62 to 6.44 ppm), a downfield shift (0.11 ppm) of 1^4^-H (from 6.88 to 6.99 ppm), and a downfield shift (0.08 ppm) of 1^6^-H (from 6.78 to 6.86 ppm). These chemical shift changes are in agreement with the predicted values [15] of closely related compounds, as will be shown below.

The following signals of Figure 5 could also be assigned to 1^4^,6^4^-dibromo-2,5,10,13-tetraoxa-7,16-diaza-1(1,2),6(1,2)-benzenecyclohexadecaphane: δ 6.44 (d, *J* = 8.7 Hz, 2H, 1^6^-H, 6^6^-H), 6.86 (d, *J* = 2 Hz, 2H, 1^3^-H, 6^3^-H), 6.99 (dd, *J* = 2 and 8.7 Hz, 2H, 1^5^-H, 6^5^-H). Comparison of these spectral data with those of the diamine **V** reveals that bromination *para*- to the oxygen group results in a downfield shift (0.24 ppm) of 1^3^-H (from 6.62 to 6.86 ppm), downfield shift (0.31 ppm) of 1^5^-H (from 6.68 to 6.99 ppm), and an upfield shift (0.34 ppm) of 1^6^-H (from 6.78 to 6.44 ppm). These chemical shift changes are different from the predicted values of closely related compounds, as discussed below.

The predicted chemical shifts of aromatic protons of 2-methoxy-*N*-methylaniline are δ 6.31, 6.47, 6.55, and 6.60 ppm for 6-H, 4-H, 3-H, and 5-H, respectively. The predicted chemical shifts of the aromatic protons of 4-bromo-2-methoxy-*N*-methylaniline are δ 6.20, 6.72, and 6.77 ppm for 6-H, 3-H, and 5-H, respectively. These data indicate that *para*-bromination of the amino group results in an upfield shift (0.11) of 6-H, a downfield shift (0.17 ppm) of 3-H, and a downfield shift (0.17) of 5-H. These chemical shift changes are close to those of the corresponding protons in compound **X**.

The predicted chemical shifts of the aromatic protons of 5-bromo-2-methoxy-*N*-methylaniline are δ 6.43, 6.48, and 6.64 ppm for 3-H, 6-H, and 4-H, respectively. These data indicate that bromination *para*- to the oxygen group results in an upfield shift (0.12 ppm) of 3-H, a downfield shift (0.17) of 6-H, and a downfield shift (0.17) of 4-H. These chemical shift changes are different than those of 1^4^, 6^4^-dibromo derivative indicating bromination of the diamine V proceeds via *para*-bromination of the amino group.

## Experimental

### General

All purifications by flash chromatography were performed on silica gel 230-400 mesh (Merck). Thin layer chromatography (TLC) was performed on silica gel IB-F (J.T. Baker). ^1^H-NMR spectra were recorded on either Varian 300 MHz or Bruker 400 MHz spectrometers. Resonances are reported as (solvent); δ = shift in ppm from tetramethyl silane (TMS) at 0 ppm and the multiplicity of signals as s, singlet; d, doublet; dd, double doublet; t, triplet; m, multiplet; br, broad; *J* values are in Hz. The numbering system used is shown in the schemes. IR spectra were measured on Nicolet FT-IR Impact 400D as KBr discs. UV-Visible spectra were obtained on a Hitachi U2000 spectrometer. FAB mass spectra were carried out using *m*-nitrobenzyl alcohol as matrix. Melting points were measured on a Laboratory Devices (Cambridge, MA) Mel-Temp apparatus and are uncorrected. High dilution reactions of acid chlorides with amines were carried out using infusion pump model 975 from Harvard Apparatus (MA, USA).

*1,2-Bis (2-nitrophenoxy)ethane* (**II**): A mixture of 2-nitrophenol (13.3 g, 96 mmol), 1,2-dibromoethane (10 g, 53 mmol) and K_2_CO_3_ (15.2 g, 110 mmol) in dimethylformamide (50 mL) was refluxed overnight. Additional 1,2-dibromoethane (10 g) was added and then refluxed for 2 hrs. The reaction was cooled to room temperature and water (50 mL) was added and the mixture stirred for 30 minutes. The resulting light brown precipitate was collected, washed with water, and recrystallized from acetone to afford yellow crystals (10.4 g, 71%); m.p.163-165 °C; IR (KBr): υ_max_ 1,606, 1,525, 1,490, 1,445, 1,364, 1,254, 1,163, 937, 858, 777, 752 cm^-1^; ^1^H-NMR (DMSO-d_6_): δ 4.52 (s, 4H, OCH_2_CH_2_O), 7.13 (t, *J* = 7.4, 2H, 4-H), 7.42 (d, *J* = 8.4, 2H, 6-H), 7.64 (dt, *J* = 1.6 and 8.0 Hz, 2H, 5-H), 7.84 (dd, *J* = 1.8 and 8.4 Hz, 2H, 3-H). 

*1,2-Bis (2-aminophenoxy)ethane* (**III**): A suspension of II (14.7 g, 48 mmol), 10% palladium on carbon (0.7 g) in methanol (400 mL) and ethyl acetate (50 mL) was hydrogenated overnight at atmospheric pressure and room temperature. The reaction mixture was filtered and the solvent removed in vacuo leaving a white solid, which was recrystallized from ethyl acetate to yield white crystals (9.5 g, 81%), m.p. 122-125 °C; IR (KBr): υ_max_ 3,446 and 3,361 (NH), 1,610, 1,504, 1,461, 1,275, 1,213, 1,082, 947, 746, 737 cm^-1^; ^1^H-NMR (DMSO-d_6_): δ 4.26 (s, 4H, OCH_2_CH_2_O), 4.68 (s, 4H, NH_2_), 6.5 (dt, *J* = 2.0 and 8.0 Hz, 2H, 5-H), 6.65 (dt, *J* = 1.6 and 8.5 Hz, 2H, 4-H), 6.70 (dd, *J* = 1.2 and 7.4 Hz, 2H, 3-H), 6.85 (dd, *J* = 1.0 and 7.6 Hz, 2H, 6-H).

*2,5,10,13-Tetraoxa-7,16-diaza-1(1,2),6(1,2)-benzacyclohexadecaphane-8,15-dione* (**IV**): To a two liter three necked flask was added a solution of triethylamine (3.5 g, 35 mmol) in anhydrous toluene (400 mL) maintained at 0 °C with stirring. The bisamine (III) (3.06 g, 12.5 mmol) in THF (150 mL) and the diacid chloride, 2,6-dioxaoctanedioic acid chloride (2.69 g, 12.5 mmol) in toluene (150 mL) were added dropwise simultaneously through Teflon syringe needles (16 gauge, 24 inch length with KEL-Fluerhub) attached to 50 mL syringes which were controlled by an infusion pump (Harvard Apparatus, model 975). The addition proceeded at a rate of 0.3 mL/min. After the addition was complete, stirring was continued for a further two days at room temperature and the reaction mixture was filtered to remove the salts. Removal of the solvent *in vacuo* left a white solid which was recrystallized from ethyl acetate to afford white crystals of the diamide **IV** (3.1 g, 64%), m.p. 183-187 °C; IR (KBr): υ_max_ 3,372, 3,356, 1,648, 1,607, 1,596, 1,460, 1,250, 1,121, 748 cm^-1^; ^1^H-NMR: (DMSO-d_6_) δ 3.80 (s, 4H, 11-CH_2_, 12-CH_2_), 4.11 (s, 4H, 9-CH_2_, 14-CH_2_), 4.47 (s, 4H, 3-CH_2_, 4-CH_2_), 6.93 (t, *J* = 6.5 Hz, 2H, 1^4^-H, 6^4^-H), 7.05 (t, *J* = 7.6 Hz, 2H, 1^5^-H, 6^5^-H), 7.90 (d, *J* = 8.0 Hz, 2H, 1^6’^-H, 6^6^-H), 8.4 (d, *J* = 7.4 Hz, 2H, 1^3^-H, 6^3^-H), 9.5 (s, 2H, -NH).

*2,5,10,13-Tetraoxa-7,16-diaza-1(1,2),6(1,2)-benzacyclohexadecaphane* (**V**): To the diamide **IV** (0.4 g, 1.0 mmol) was added LiAlH_4_ in THF (1.0 M, 10 mL) dropwise with stirring at room temperature. The reaction mixture was heated under reflux overnight. The excess of LiAlH_4_ was destroyed by the successive dropwise addition of water (0.4 mL), NaOH (15%, 0.4 mL) and water (1.2 mL). The reaction mixture was filtered and the filtrate was evaporated to dryness to leave a solid which was recrystallized from ethyl acetate/hexane to afford white crystals (0.24 g, 67%), m.p. 165-167 °C; IR (KBr): υ_max_ 3,422, 1,597, 1,523, 1,448, 1,253, 1,222, 1,120, 941, 733, 715 cm^-1^; ^1^H-NMR (CDCl_3_): δ 3.33 (m, 4H, 8-CH_2_, 15-CH_2_), 3.60 (s, 4H, 11-CH_2_, 12-CH_2_), 3.71 (t, *J* = 4.7 Hz, 4H, 9-CH_2_, 14-CH_2_), 4.38 (s, 4H, 3-CH_2_, 4-CH_2_), 4.80 (br s, 2H, NH), 6.62 (dd, *J* = 1.43 and 7.80 Hz, 2H, 1^3^-H, 6^3^-H), 6.68 (dt, *J* = 1.53 and 7.70 Hz, 2H, 1^5^-H, 6^5^-H), 6.78 (dd, *J* = 1.4 and 7.95 Hz, 2H, 1^6^-H, 6^6^-H), 6.88 (dt, *J* = 1.40 and 7.62 Hz, 2H, 1^4^-H, 6^4^-H).

*4,7,13,16,20,23-Hexaoxa-1,10-diaza-19(1,2)24(1,2)-dibenzabicyclo[8.8.6]tetracosaphane-2,9-dione* (**VI**): To a stirred solution of triethylamine (2.61 g, 25.8 mmol) in toluene (500 mL) maintained at 0 °C was added the bisamine **V** (3.0 g, 8.4 mmol) in THF (200 mL) and the diacid chloride, 3,6-dioxaoctanedioic acid chloride (2.1 g, 9.8 mmol) in toluene (200 mL), simultaneously using 50 mL syringes. The rate of addition (0.3 mL/min) was controlled by an infusion pump. After addition was completed, the mixture was stirred at room temperature overnight. Removal of the solvent *in vacuo* left a semisolid which was crystallized from ethyl acetate to give white crystals (1.6 g, 40%), m.p. 186-189 °C; IR (KBr): υ_max_ 1,672, 1,499, 1,452, 1,275, 1,132, 933, 755 cm^-1^; ^1^H-NMR (CDCl_3_): δ 3.05-4.75 (m, 24H), 6.96-7.16 (m, 6H), 7.25-7.40 (m, 12H). 

*4,7,13,16,20,23-Hexaoxa-1,10-diaza-19(1,2)24(1,2)-dibenzabicyclo[8.8.6]tetracosaphane* (**VII**): To a solution of diamide VI (0.2 g, 0.4 mmol) in THF (2 mL) was added borane in THF (1 M, 2 mL, 2 mmol), and the mixture was heated under reflux for 2 hrs, cooled and water (0.5 mL) carefully added followed by aqueous HCl (6 M, 4 mL). The solution was concentrated *in vacuo* and the resulting yellow solution was brought to pH 8 with aqueous LiOH. The aqueous layer was extracted with chloroform and dried over sodium sulfate. Removal of the solvent yielded a yellow oil which was triturated with ethyl acetate to give a white powder, m.p.141-143 °C; IR (KBr): υ_max_ 1,593, 1,505, 1,449, 1,341, 1,250, 1,230, 1,135, 1,115, 1,056, 975, 940, 732 cm^-1^ ; ^1^H-NMR (CDCl_3_): δ 3.12 (m, 4H), 3.54 (m, 16H), 3.81 (m, 4H), 4.37 (s, 4H, 2CH_21_, 2CH_22_), 6.92 (m, 8H, aromatic).

*19^5^,24^5^-Dibromo-4,7,13,16,20,23-hexaoxa-1,10-diaza-19(1,2)24(1,2)-dibenzabicyclo[8.8.6]tetra-cosaphane* (**VIII**): To a stirred solution of diamine **VII** (116 mg, 0.25 mmol) in CH_2_Cl_2_ (5 mL) kept at -78 °C was added portion-wise 2,4,4,6-tetrabromo-2,5-cyclohexadien-1-one (0.25 g, 0.61 mmol). The mixture was allowed to warm to room temperature and stirred overnight. The resulting yellow solution was extracted with aqueous NaOH (2 N, 10 mL), water and the organic layer was dried over sodium sulfate. Removal of the solvent afforded a yellow oil which was chromatographed using silica gel and methylene chloride and methanol (up to 5%) to give white solid which was recrystallized from ethyl acetate to yield white crystals (31 mg, 20 %), m.p. 204-206 °C; IR (KBr): υ_max_ 1,580, 1,489, 1,232, 1,125, 959, 848, 817 cm^-1^; ^1^H-NMR (CDCl_3_): δ 3.09-3.16 (m, 4H), 3.36-3.42 (m, 4H), 3.46-3.52 (m. 4H), 3.58-3.66 (m, 8H), 3.76-3.83 (m, 4H), 4.48 (s, 4H, 2CH_21_, 2CH_22_), 6.78 (d, *J* = 8.0 Hz, 2H, 19^3^-H, 24^3^-H), 7.04 (dd, *J* = 8.0 and 2.4 Hz, 2H, 19^4^-H, 24^4^-H), 7.06 (d, *J* = 2.4 Hz, 2H, 19^6^-H, 24^6^-H); *m/z* (FAB) 651 (M+Na^+^).

*1^5^-Bromo-2,5,10,13-tetraoxa-7,16-diaza-1(1,2),6(1,2)-benzenecyclohexadecaphane* (**IX**): To a stirred solution of diamine **V** (150 mg, 0.4 mmol) and pyridine (98 mg, 1.2 mmol) in CH_2_Cl_2_ (10 mL) and kept at -78 °C was added slowly 0.78 M bromine solution in CH_2_Cl_2_ (1.3 mL). The mixture was allowed to warm to room temperature. The reaction mixture was washed with aqueous Na_2_CO_3_ and dried over sodium sulfate. Removal of the solvent yielded a residue which was chromatographed on a TLC plate (200 µm layer of silica gel IB-F, 1% CH_3_OH in CH_2_Cl_2_ as solvent) to give two major products: the monobromo-derivative **IX** (22 mg, 13%), m.p. 158-161 °C; IR (KBr): υ_max_ 3,405, 1,603, 1,520, 1,453, 1,261, 1,202, 1,141, 1,096, 942, 838, 799, 724 cm^-1^; ^1^H-NMR (CDCl_3_): δ 3.31 (m, 4H, 8-CH_2_, 15-CH_2_), 3.60 (s, 4H, 11-CH_2_, 12-CH_2_), 3.70 (m, 4H, 9-CH_2_, 14-CH_2_), 4.36 (m, 4H, 3-CH_2_, 4-CH_2_), 4.76 (br s, 2H, NH), 6.44 (d, *J* = 8.8 Hz, 1H, 1^3^-H), 6.61 (d, *J* = 7.5 Hz, 1H, 6^3^-H), 6.68 (t, *J* = 7.5 Hz, 1H, 6^5^-H), 6.78 (d, *J* = 7.5 Hz, 1H, 6^6^-H), 6.86 (d, *J* = 2.0 Hz, 1H, 1^6^-H), 6.90 (t, *J* = 7.5 Hz, 1H, 6^4^-H), 6.98 (dd, *J* = 2 and 8.7 Hz, 1H, 1^4^-H); and the dibromo derivative **X** (20 mg, 10%), m.p. 210-214 °C; IR (KBr): υ_max_ 3,405, 1,598, 1,509, 1,463, 1,406, 1,346, 1,217, 1,203, 1,146, 1,097, 939, 843, 794 cm^-1^; ^1^H-NMR (CDCl_3_): δ 3.29 (q, *J* = 5 Hz, 4H, 8-CH_2_, 15-CH_2_), 3.60 (s, 4H, 11-CH_2_, 12-CH_2_), 3.69 (t, *J* = 5 Hz, 4H, 9-CH_2_, 14-CH_2_), 4.33 (s, 4H, 3-CH_2_, 4-CH_2_), 4.73 (br t, *J* = 5 Hz, 2H, NH), 6.44 (d, *J* = 8.7 Hz, 2H, 1^3^-H, 6^3^-H), 6.86 (d, *J* = 2 Hz, 2H, 1^6^-H, 6^6^-H), 6.99 (dd, *J* = 2 and 8.7 Hz, 2H, 1^4^-H, 6^4^-H). 

*1^5^,6^5^-Dibromo-2,5,10,13-tetraoxa-7,16-diaza-1(1,2),6(1,2)-benzenecyclohexadecaphane* (**X**): To a stirred solution of of diamine **V** (1.4 g, 4 mmol) in CH_2_Cl_2_ (50 mL) maintained at -78 °C was added finely powdered 2,4,4,6-tetrabromo-2,5-cyclohexadien-1-one (3.4 g, 8 mmol). After addition was completed, the reaction mixture was allowed to warm to room temperature and stirred overnight. The resulting yellow solution was extracted with aqueous 2N sodium hydroxide. The organic layer was washed with water and dried over sodium sulfate. Removal of the solvent afforded a yellow solid which was recrystallized from ethyl acetate to yield white flakes (1.6 g, 78%), m.p. 212-215 °C, ^1^H-NMR spectral data were identical to those given above.

*19^5^,24^5^-Dibromo-4,7,13,16,20,23-hexaoxa-1,10-diaza-19(1,2)24(1,2)-dibenzabicyclo[8.8.6]tetra-cosaphane-2,9-dione* (**XI**): To a stirred solution of triethylamine (1.45 g, 14.3 mmol) in toluene (300 mL) maintained at 0 ^ο^C were added the bisamine **X** (1.4 g, 2.7 mmol) in THF (100 mL), and 3,6-dioxa-octanedioic acid chloride (0.6 g, 2.8 mmol) in toluene (100 mL), simultaneously at a rate of 0.3 mL/min with the aid of an infusion pump. After the addition was completed, the mixture was stirred at room temperature overnight. The mixture was filtered and removal of solvent *in vacuo* gave an oil which was chromatographed using silica and CH_2_Cl_2_ and methanol (10%) to yield a white solid (0.27 g,15%), m.p. 282-289 ^ο^C (decomposition); IR (KBr): υ_max_1,684, 1,586, 1,494, 1,403, 1,273, 1,134, 1,102, 952, 853, 739 cm^-1^; ^1^H-NMR (CDCl_3_): δ 3.25-4.75 (m, 24H), 6.94-7.03 (m, 2H), 7.10-7.20 (m, 4H).

*Conversion of* **XI** *to* **VIII**: To a solution of diamide XI (200 mg, 0.3 mmol) in THF (4 mL) was added borane in THF (1 M, 3 mL), and the mixture heated under reflux for 2 hrs. The reaction mixture was worked up as described above to yield white solid, which showed IR (KBr) and ^1^H-NMR data similar to those of **VIII** reported above.

## Conclusions

In summary, the dibromdibenzocryptand [2.2.2] **VIII**, a potential potassium ionophore, has been synthesized. The bromo derivatives of the benzocryptand are likely be linked to a fluorophore, such as those described by McGimpsey in [16].
